# Design of a Low-Infrared-Emission and Wideband-Microwave-Absorption Lightweight Metasurface

**DOI:** 10.3390/nano15050399

**Published:** 2025-03-05

**Authors:** Liping Liu, Zongsheng Chen, Zhigang Li, Yajing Chang, Pengfei Li, Xun Liu, Xuesong Deng, Yunsong Feng

**Affiliations:** 1 Advanced Laser Technology Laboratory of Anhui Province, College of Electronic Engineering, National University of Defense Technology, Hefei 230037, China; liuliping10@nudt.edu.cn (L.L.);; 2 School of Mechanical Engineering, Jiangsu University, Zhenjiang 212013, China; 3 The Information Materials and Intelligent Sensing Laboratory of Anhui Province, Anhui University, Hefei 230039, China

**Keywords:** infrared–radar-compatible stealth technology, metasurface, light weight

## Abstract

The compatibility of low infrared emission and wideband microwave absorption has drawn extensive attention, both theoretically and practically. In this paper, an infrared–radar-compatible stealth metasurface is designed using transparent conductive materials, namely indium tin oxide (ITO) and poly methacrylimide (PMI). The designed structure is a combination of a radar-absorbing layer (RAL) and a low-infrared-emission layer (IRSL), with an overall thickness of about 1.7 mm. It consists of three layers, a top-layer patch-type ITO frequency-selective surface, an intermediate layer of a four-fold rotationally symmetric ITO patterned structure, and a bottom reflective surface. The layers are separated by PMI. Simulation results show that the structure achieves over 90% broadband absorption in the microwave band from 7 to 58 GHz and low emissivity of 0.36 in the infrared band. In addition, due to the four-fold rotationally symmetric design, the structure also exhibits polarization insensitivity and excellent angular stability. Therefore, the designed structure possesses ultra-broadband radar absorption performance, low infrared emissivity, and polarization-insensitive properties at a thin thickness, and has a promising application in the field of multi-band-compatible stealth technology.

## 1. Introduction

With the development of detection methods [1,2,3], the role of stealth technology is becoming more and more important, especially in modern information warfare. In recent years, the complexity of multispectral detection technology has increased dramatically [4,5,6], hence traditional single-band stealth technology has been insufficient to meet the challenge of multiple detection. The development of multispectral and efficiently compatible stealth materials with microwave stealth, infrared and visible-light stealth technologies has become urgent and necessary [7,8,9,10].

Infrared and radar compatible stealth technologies are particularly important and challenging because their operating mechanisms are contradictory. For radar stealth technology, the use of absorbing materials requires low reflection to avoid microwave detection [11,12,13]. On the contrary, realizing infrared stealth technology requires high reflection and low absorption (low emissivity) [14,15,16]. Determining how to solve this contradiction is the key to realizing infrared–radar-compatible stealth technology [17,18]. So far, mainstream infrared–radar-compatible stealth technology is mainly categorized into two types. One approach is to develop a single type of material with both high radar absorption and low infrared emissivity, such as conductive polymers, nanomaterials, and doped oxide semiconductors [19,20,21]. Another approach is to design composite structures by covering microwave absorbers with low-infrared-reflective layers [9,10,22]. Although perfect infrared–radar compatibility can be achieved by adjusting the material doping concentration and coating thickness, the preparation of such a single type of material requires complicated processes and equipment. The types, concentrations, and distributions of dopant ions need to be precisely controlled. In addition, the internal structure tends to be more complex and susceptible to the influence of external environment and changes. Therefore, there are still obstacles to producing such multispectral stealth materials on a large scale and at low costs.

Metamaterials have provided a new solution for realizing infrared–radar-compatible stealth technology due to their advantages such as the controllable structural design, easy integration, and flexible modulation of multispectral waves [23,24]. Meng et al. [25] proposed an ITO/dielectric/ITO sandwich structure with more than 90% microwave absorption in the 8–18 GHz range, with an infrared emissivity of about 0.36 in the 3–14 μm range, and with a thickness of only 2.4 mm. Wang et al. [26] reported fan-shaped, box-nested, silver nanowire graphene super-surfaces with an ultra-low infrared emissivity and broadband and high absorption properties in the 10–37.5 GHz region, with a thickness of about 3 mm. Gao et al. [27] designed a multilayer structure that realizes microwave absorption in the range of 1.81–20.4 GHz with low infrared emissivity of 0.271, but a thickness of up to 10.34 mm. However, the above structures inevitably show increasing thickness when realizing broadband microwave absorption, which greatly restricts its application; thus, further research and development are urged.

In this paper, an infrared–radar-compatible stealth structure is proposed. The lightweight structure is made of transparent conductive materials of ITO and PMI. Through reasonable structural design, lightweight broadband-wave-absorbing characteristics as well as low infrared emissivity are realized. Our work presents a new solution for competitive infrared–radar stealth technology that is prospectively applicable in the increasing complex battlefield.

## 2. Structural Design

**Figure 1:**
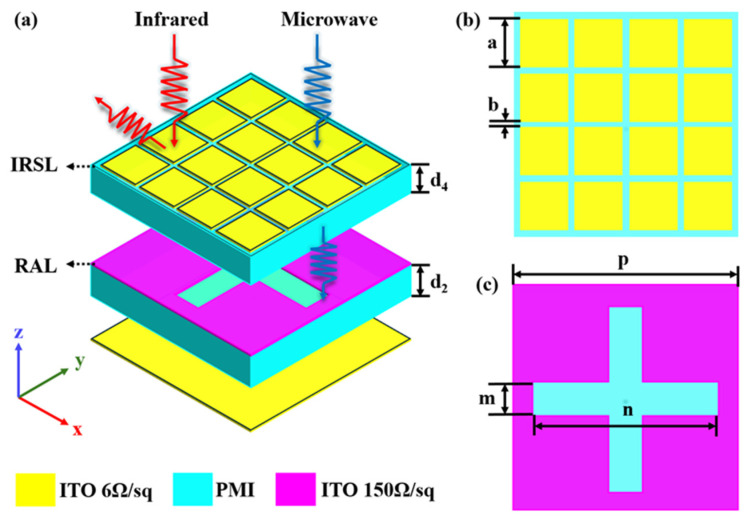
Schematic diagram of the unit structure. (**a**) front view of absorber; (**b**) top view of IRSL; (**c**) top view of RAL.

Since infrared and radar stealth technologies have different material and structural requirements, the infrared and radar stealth structures are designed separately and then combined to achieve infrared–radar stealth compatibility. The unit structure is designed, as shown in Figure 1a, including IRSL and RAL layers. A specific introduction is discussed below.

The infrared stealth layer is designed to fulfill two conditions. On one hand, it should have low emissivity in the infrared band. On the other hand, the layer should allow effective penetration of electromagnetic waves in the radar band. An effective way to achieve infrared stealth properties is to reduce infrared radiation. Metals have a relatively low emissivity, but the reflectivity increases dramatically with increasing temperature. To solve this problem, ITO films are used as low-infrared-reflective layers. ITOs exhibit transparency in the visible spectral range, while in the infrared band, their dielectric constant satisfies the Drude model [28]:

(1) $$\varepsilon \left(\omega \right)=\varepsilon_{b}-\frac{\omega_{p}^{2}}{\omega \left(\omega +i\omega_{c}\right)}$$

where the dielectric constant $\varepsilon_{b}=3.9$, plasma frequency $\omega_{p}=461\;THz$, and collision frequency $\omega_{c}=28.7\;THz$. Therefore, ITO has a negative real part of the dielectric constant in the infrared band, and metal-like properties of low emissivity. However, ITO has better oxidation resistance than metal. Modifying the sheet resistance of an ITO film can change its electrical conductivity, thereby affecting its ability to reflect and absorb infrared light. Higher sheet resistance (lower thickness) results in stronger reflectivity and lower absorptivity, which leads to lower infrared emissivity [29,30,31]. The continuity of an ITO film, which ensures uniform electrical conductivity, can bring an excellent infrared stealth effect by reducing infrared emissivity [32]. However, when its thickness is increased to close to the skin depth, a significant reflection phenomenon will occur. Therefore, a capacitive frequency-selective surface with low-pass filtering characteristics is used to realize high microwave transmittance. The capacitive frequency-selective surface is meant to fulfill the infrared–radar-compatible stealth requirement. Thus, it should have low reflectivity in the radar band and low emissivity in the infrared band, which means high reflectivity in the infrared band. The structure is shown in Figure 1b, which is a top view of the IRSL, consisting of a periodic arrangement of ITO patches with edge lengths, *a*, and patch gaps, *b*. Based on the standing wave theory, the resonant frequency of the periodic patch satisfies [33].

(2) $$f=\frac{c}{2\sqrt{\varepsilon_{r}}a}$$

where $c=3\times 10^{8}\;m/s$; $\varepsilon_{r}$ is the dielectric constant of the material. According to formula (2), it is found that the smaller the size of the ITO patch, the higher its corresponding resonant frequency and microwave transmittance.

As is shown in Figure 1a, the proposed infrared–radar-compatible stealth structure selects the optically transparent ITO and PMI, with a PMI dielectric constant of 1.12 (1 − 0.01i). The top layer is the IRSL with ITO square resistance of $R_{1}=6\;\Omega /sq$. The bottom layer is the RAL layer. The top view is shown in Figure 1c. It is composed of an ITO–PMI–ITO sandwich structure, and the ITO square resistance of the top frequency-selective surface is $R_{1}=150\;\Omega /sq$. The square resistance of the bottom ITO back panel is 6 Ω/sq. The structural parameters are optimized based on the theory of material electromagnetic characteristics. We carried out a series of adjustments and tests on the structural parameters using the parameter scanning method. In this process, we took into account multiple factors, including material properties, as well as the requirements for infrared and radar stealth performance. The optimized structure parameters of the IRSL and ITO backplane are as follows:$\;d_{1}=175\;nm$, $d_{2}=1\;mm$, $\;d_{3}=0.02\;mm$, $\;d_{4}=0.7\;mm$,  a=0.8 mm,  b=0.2 mm, p=10 mm,m=1mm,and n=9mm. The total thickness of the structure is about 1.7 mm.

## 3. Results and Discussion

**Figure 2:**
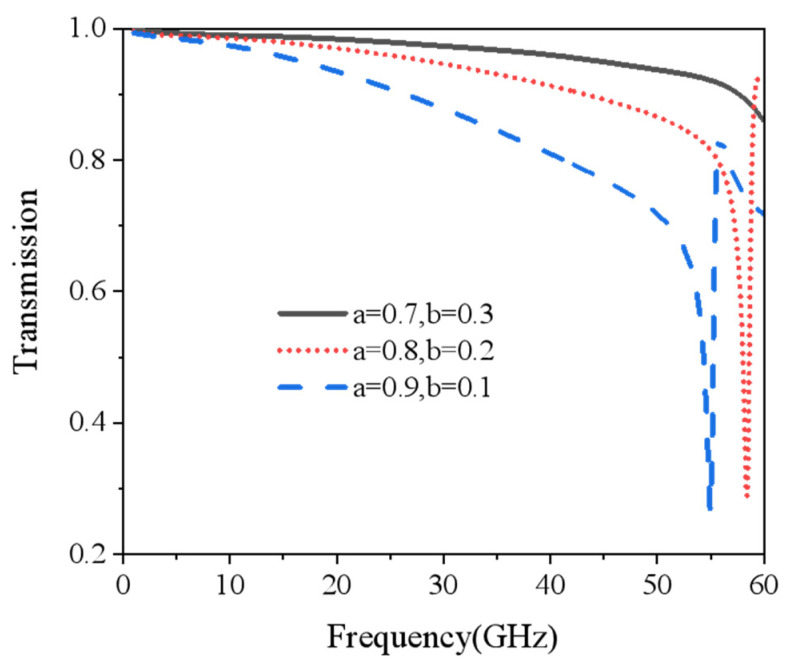
Microwave transmittance of IRSL with different patch sizes.

The commercial electromagnetic software CST Microwave Studio was utilized for modeling and simulation. The boundary conditions in the x and y directions were set as the unit cell, and the ±z direction was set as open (add space). The mesh size was adaptive tetrahedral mesh refinement. Finally, the frequency domain solver was used for simulation. As is shown in Figure 2, the microwave transmittance of IRSL with different sizes of ITO patches were calculated. It was found that the smaller the size of the patch, the higher the resonance frequency and the electromagnetic wave transmittance. When the ITO patch edge length was 0.8 mm, the microwave transmittance below the resonant frequency was greater than 0.8. The total emissivity of the IRSL can be expressed as [34]:

(3) $$\varepsilon =\varepsilon_{ITO}f_{ITO}+\varepsilon_{PMI}\left(1-f_{ITO}\right)$$

$\varepsilon_{ITO}$ and $\varepsilon_{PMI}$ indicate the emissivity of ITO and the substrate PMI used for etching ITO, respectively. $f_{ITO}$ is the duty cycle of ITO, namely the ITO occupied area/total area. As can be seen from Formula (3), the larger the ITO duty cycle, the lower the infrared emissivity. Meanwhile, the high microwave transmittance should also be taken into account. Therefore, the ITO patch size was selected as follows: *a* = 0.8 mm and *b* = 0.2 mm. As a result, the estimated IRSL emissivity was approximately 0.36.

In order to obtain the highest possible microwave absorbance, the reflection coefficient needed to be minimized. The reflection coefficient Γ was obtained from the following equation [35]:

(4) $$\Gamma =\frac{Z_{in}-\eta_{0}}{Z_{in}+\eta_{0}}$$

where $Z_{in}$ is the input impedance of the metasurface and $\eta_{0}$ is the free space characteristic impedance. $Z_{in}$ is closely related to the sheet resistance of the pattern structure, the pattern shape, the dielectric substrate material, and the dielectric substrate thickness. Rationally designing and optimizing the geometrical parameters of the absorber, the input impedance $Z_{in}$ can be maximally matched with the free-space characteristic impedance $\;\eta_{0}$. In this way, we can successfully obtain the minimum reflection coefficient.

**Figure 3:**
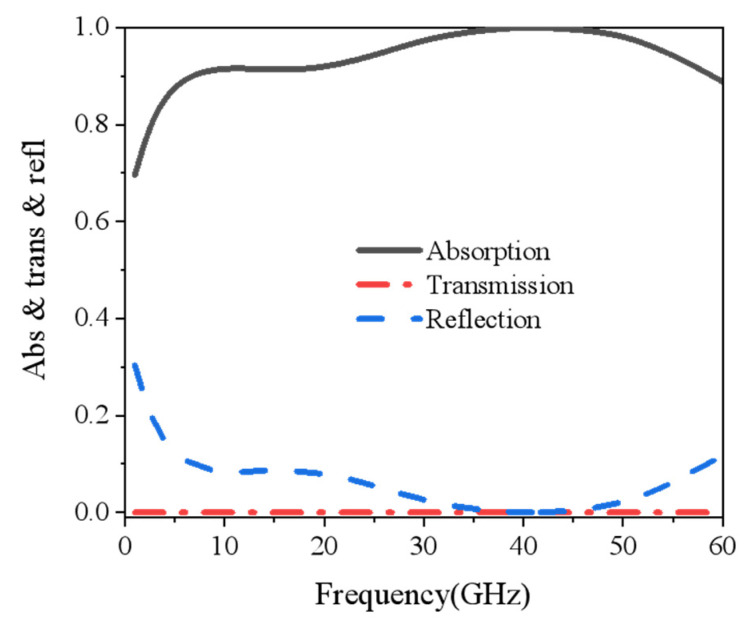
Absorption, transmittance, and reflectance simulation structure.

Figure 3 shows the calculation results of reflectance, transmittance, and absorptance of the electromagnetic wave when it is vertically incident on the metasurface. The black solid line, red dotted line, and blue dashed line denote the absorption, transmission, and reflection coefficient, respectively. Since the bottom of the metasurface has an ITO back panel with a square resistance of 6 Ω/sq, which can almost completely reflect microwaves and block almost all microwave transmissions, the transmittance rate was approximated to be 0. The absorption of the metasurface can be simplified as following

(5) A=1−R

where $R={\left|S_{11}\right|}^{2}$. As is shown by the black solid line in Figure 3, the structure achieves more than 90% absorption in the 7–58 GHz broadband. In addition, the metasurface achieves around 100% absorption at 41 GHz. In this case, the reflection coefficient Γ is approximated to be zero, and it can be assumed that the input impedance of the metasurface $Z_{in}$ matches well with the characteristic impedance of free space $\eta_{0}$.

**Figure 4:**
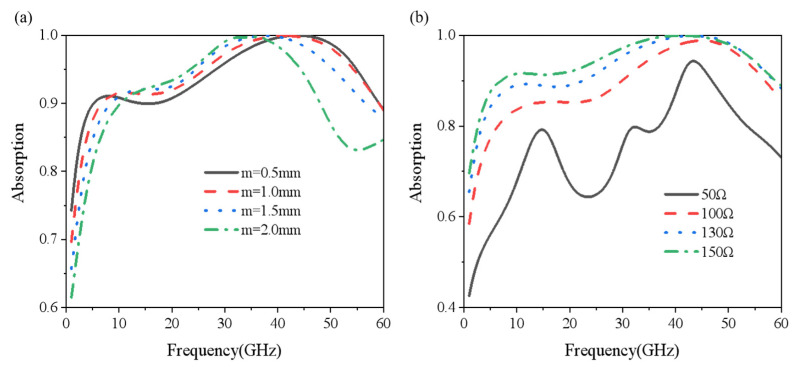
(**a**) Absorbance at different patch sizes; (**b**) absorbance at different square resistances.

Figure 4a indicates the effect of the cross-seam width of the RAL layer on the radar absorption performance as the size of the cross-seam width m changing from 0.5 mm to 2 mm. As can be seen, with the increase in the seam width m, the absorption at a low frequency increases gradually and blue shifts. At a high frequency, the absorption is almost unchanged. On the whole, the absorption bandwidth becomes narrower as the gap width increases. Similarly, Figure 4b shows the absorption with the square resistance of the ITO in the RAL layer changing from 50 Ω to 150 Ω. The gap width was set to 1 mm and other parameters remained unchanged. With the increase in the square resistance of ITO, the overall absorption also increased, which was caused by the larger loss of the ITO resistance.

**Figure 5:**
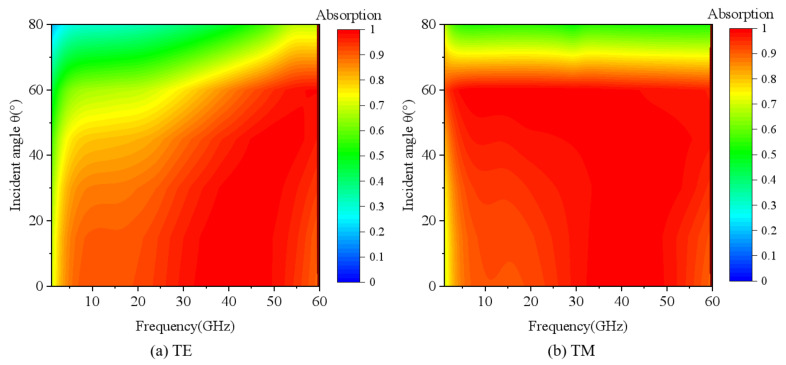
Absorbance at different incidence angles for (**a**) TE and (**b**) TM modes.

Figure 5 shows the absorption performance of the infrared–radar-compatible stealth structure at different polarization waves and incidence angles. The structure is polarization insensitive due to the high symmetry of the designed structure. Keeping other parameters unchanged and increasing the incidence angle from 0° to 80°, Figure 5a shows the absorption curves at different incidence angles in the TE mode. As can be seen, the absorption rate at a low frequency decreases gradually with the increase in the incidence angle, while the absorption rate at the high frequency remains almost constant. When the incidence angle is increased to 45°, the proposed compatible stealth structure still maintains nearly 80% absorption in the operating frequency band. Figure 5b shows the absorption curves for different incidence angles in the TM mode. When the incidence angle is increased to 60°, the absorption of the proposed compatible stealth structure remains above 90%. When the incidence angle is increased to 80°, the absorption starts to decrease, but there is still nearly 80% absorption at 70° oblique incidence.

**Figure 6:**
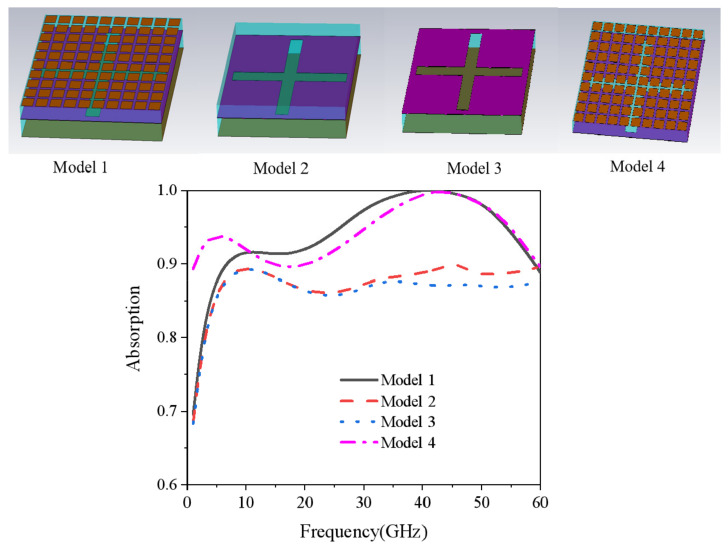
Absorption rate curves for different models.

Figure 6 shows the radar absorption performance of different structures of Model 1 to Model 4. Model 1 is the proposed compatible stealth structure. Model 2 is the structure with the top IRSL removed, and Model 3 is the typical ITO–dielectric–ITO absorbing structure with the top IRSL and PMI dielectric removed. The relevant structural parameters were kept constant for all three parts, and the absorptivity curves obtained from the simulation of each part are shown in Figure 6. From the blue dotted line of the absorption curve of Model 3, it is easy to see that the overall absorption rate of Model 3 is higher but not up to 90%. Model 2 is similar to Model 3, and only the absorption rate at the high frequency is slightly improved, which is due to the small effect of the dielectric loss of PMI on the absorption rate. Comparing the absorption rates of Model 1 and Model 2, the overall absorption rate of Model 2 is greatly improved, covering the frequency-selective surface of the patch type. In order to better analyze the influence of the frequency-selective surface of the patch type on the absorption rate, the IRSL, the PMI dielectric, and the frequency-selective surface of the ITO of the cross slit were composed into Model 4. The simulated structure is shown as the pink dotted–dashed line in Figure 6. The absorption of Model 4 was mostly over 90% in the operating frequency band, and only a little bit lower at around 20 GHz. In comparison with Model 1, the absorption of Model 4 at high frequencies is mainly caused by the interaction between the patch-type frequency-selective surfaces and the frequency-selective surfaces with cross slits, while at low frequencies it is caused by the interaction between the reflective back panel and the frequency-selective surfaces with cross slits.

**Figure 7:**
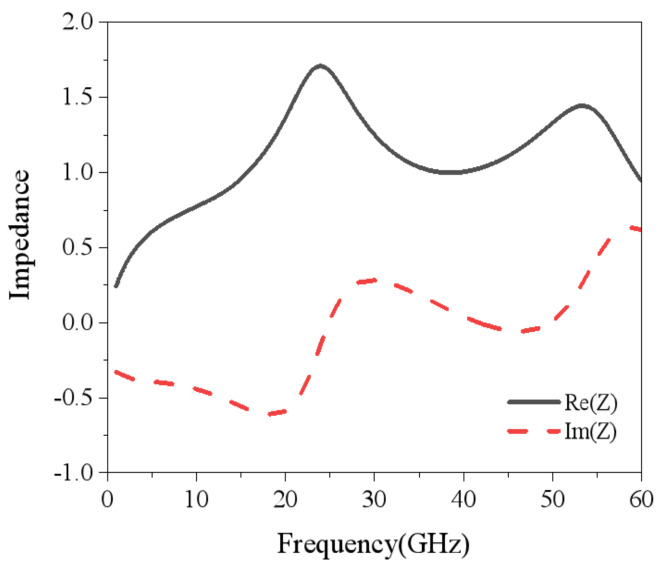
The real part and imaginary part of the characteristic impedance.

In order to investigate the absorption mechanism of the presented structure, the equivalent parameter was analyzed firstly. For the periodic array structure, the equivalent medium theory was used to invert the equivalent parameter of the metasurface. The characteristic impedance of the metasurface can be obtained from Equation (6) [36]:

(6) $$Z_{eff}=\sqrt{\frac{{\left(1+S_{11}\right)}^{2}-S_{21}^{2}}{{\left(1-S_{11}\right)}^{2}-S_{21}^{2}}}$$

where $S_{11}$ and $S_{21}$ denote the reflection and transmission coefficients of the metasurface, respectively. Figure 7 depicts the characteristic impedance of this infrared–radar-compatible stealth structure. The black solid line is the real part of the characteristic impedance and the red dashed line is the imaginary part of the impedance. The real part fluctuates near 1 while the imaginary part fluctuates near 0 in the range of 7–58 GHz. This indicates that the infrared–radar-compatible stealth structure achieves a good match in the free space of the operating frequency band. Meanwhile, at 41 GHz, the real part is approximated to be 1 while the imaginary part is approximated to be 0; hence, near-perfect absorption is realized.

**Figure 8:**
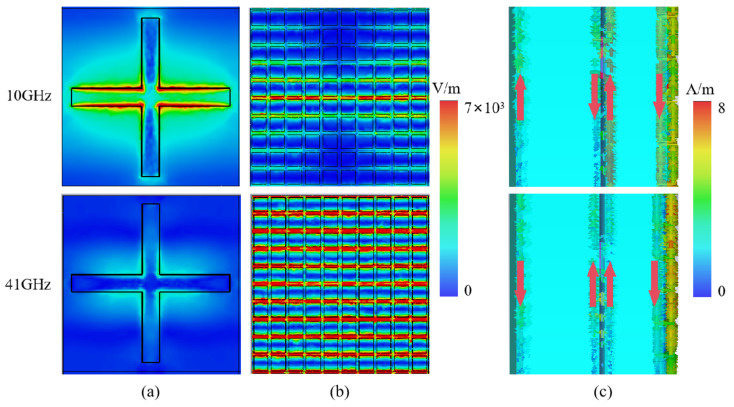
Electric field distribution at 10 GHz and 41 GHz, and surface current distribution. (**a**) Electric field distribution in RAL layer; (**b**) electric field distribution in IRSL; (**c**) surface current distribution.

In order to further reveal the mechanism of infrared–radar-compatible stealth structure absorption, the electric field distribution and current distribution of the absorber were studied at low frequency (10 GHz) and high frequency (41 GHz), as shown in Figure 8.

Figure 8a and Figure 8b show the electric field intensity distribution at the frequency-selective surface of the cross slit of the RAL layer and the frequency-selective surface of the patch type of the IRSL, respectively. At 10 GHz, the electric field is mainly concentrated inside the cross slit of the RAL layer and the patch slit of the IRSL in the corresponding position. Figure 8c shows the side view of the surface current distribution of the overall structure. The direction of the current on the lower surface of the intermediate-layer frequency-selective surface is opposite to the direction of the current on the reflective backplane, forming a current loop. However, the current distribution generates a strong magnetic resonance, which effectively absorbs the electromagnetic energy. At the same time, a current loop comes out to form a magnetic resonance between the intermediate- and the top-patch frequency-selective surface. Thus, the strong absorption at low frequency absorption is mainly caused by the strong electric resonance excited by the cross slit of the intermediate frequency-selective surface and the strong magnetic resonance excited between the ITO surfaces. At 41 GHz, the electric field is mainly concentrated in the gap between the patch-type surfaces. The whole structure also generates two current loops. Therefore, the strong absorption at high frequencies is mainly caused by the strong electric resonance excited between the small patches of the IRSL as well as the strong magnetic resonance excited between the ITO surfaces.

Table 1 compares the performance of the proposed structures in the manuscript with other metasurfaces reported. The proposed structure in this study exhibits the widest absorption bandwidth and minimum thickness. Furthermore, demonstrations are conducted on low infrared emissivity.

## 4. Conclusions

Based on the flexible transparent conductive material, ITO, as well as the dielectric material, PMI, an ultra-broadband infrared–radar-compatible stealth structure has been designed. The structure is combined with an infrared layer of low infrared emission and a radar layer of broadband radar absorption. More than 90% absorption was realized in the frequency band range of 7–58 GHz, while a low emission of 0.36 was achieved in the infrared band of 3–14 μm with a total thickness of 1.7 mm. In addition, due to the four-fold rotationally symmetric design, the structure also exhibits polarization insensitivity and excellent angular stability. Compared with previous studies, the structure is characterized by low infrared reflection, ultra-broadband radar absorption, and lightweight characteristics, and has broad application prospects in the field of infrared–radar-compatible stealth technology.

## Figures and Tables

**Table 1:** Comparison of the proposed device with those used in previous studies.

Absorbers	Absorption Bandwidth (GHz)	Relative Bandwidth (%)	Infrared Emissivity	Thickness (mm)
[37]	6–18	100	0.3	7
[38]	8.27–17.65	72.4	0.27	2.9
[39]	1.98–18.6	161.5	0.28	9.5
[40]	2.12–15.87	152.9	0.25	9
[41]	2.53∼34.56	172.7	0.23	11.5
This study	7–58	157	0.36	1.7

## Data Availability

The original contributions presented in this study are included in the article. Further inquiries can be directed to the corresponding author.
